# Genetic evaluation of test-day milk yields from smallholder dairy production systems in Kenya using genomic relationships

**DOI:** 10.3168/jds.2018-15807

**Published:** 2019-04-03

**Authors:** J. M. K. Ojango, R. Mrode, J. E. O. Rege, D. Mujibi, E. M. Strucken, J. Gibson, O. Mwai

**Affiliations:** 1 International Livestock Research Institute, Box 30709-01001 Nairobi, Kenya; 2 Scotland’s Rural College, Easter Bush, Midlothian, EH25 9RG, United Kingdom; 3 Emerge-Africa, PO Box 1450-00502, Nairobi, Kenya; 4 Usomi Limited, Hardy Post, PO Box 105086-00100, Nairobi, Kenya; 5 University of New England, Armidale 2350, Australia

**Keywords:** smallholder farm, genomic relationship, dairy production, random regression analyses

## Abstract

Efforts to improve dairy production in smallholder farming systems of East Africa over the past decade have had limited impact because of the lack of records on performance to guide targeted breeding programs. Estimates of genetic parameters in these systems are lacking. Using data generated through a project (“Germplasm for Dairy Development in East Africa”) in Kenya and a genomic relationship matrix from genotypic records, we examined the potential impact of different models handling contemporary groups or herd effects on estimates of genetic parameters using a fixed regression model (FRM) for test-day (TD) milk yields, and the covariance structure for TD milk yield at various stages of lactation for animals using a random regression model (RRM). Models in which herd groups were defined using production levels derived from the data fitted the data better than those in which herds were grouped depending on management practices or were random. Lactation curves obtained for animals under different production categories did not display the typical peak yield characteristic of improved dairy systems in developed countries. Heritability estimates for TD milk yields using the FRM varied greatly with the definition of contemporary herd groups, ranging from 0.05 ± 0.03 to 0.27 ± 0.05 (mean ± standard error). The analysis using the RRM fitted the data better than the FRM. The heritability estimates for specific TD yields obtained by the RRM were higher than those obtained by the FRM. Genetic correlations between TD yields were high and positive for measures within short consecutive intervals but decreased as the intervals between TD increased beyond 60 d and became negative with intervals of more than 5 mo. The magnitude of the genetic correlation estimates among TD records indicates that using TD milk records beyond a 60-d interval as repeated measures of the same trait for genetic evaluation of animals on smallholder farms would not be optimal. Although each individual smallholder farmer retains only a few animals, using the genomic relationship between animals to link the large number of farmers operating under specified environments provides a sufficiently large herd-group for which a breeding program could be developed.

## INTRODUCTION

Smallholder farming systems in tropical countries contribute significantly to the dairy sectors but are constrained by both technological and infrastructural challenges (Kosgey et al., 2011; Zonabend et al., 2013). In East Africa (**EA**), smallholder dairy farmers keep animals of mixed breed-types; however, productivity levels are low and the countries are not able to meet their national demands for milk production (MOLD, 2010; Majiwa et al., 2013; Makoni et al., 2013; SNV, 2013). Over the last 2 decades, there have been concerted efforts to improve dairy production in EA, with a strong focus on community development (Makoni et al., 2013; Ojango et al., 2016). The level of impact in each country has been variable because of the lack of targeted breeding programs with objectives and strategies relevant to specific production systems.

Within EA, Kenya has the largest population of dairy cattle, which are mainly reared by smallholder farmers (Thorpe et al., 2000; Moll et al., 2007; MOLD, 2010; Wanjala and Njehia, 2014), providing a livelihood to more than 1.8 million households (MOLD, 2010). Smallholders predominantly use dairy cows that are mixed-composition crossbreds of *Bos taurus* dairy breeds and indigenous zebu breeds. Evidence from studies on productivity levels achievable under smallholder systems demonstrates that cattle generated by using bulls and semen of North American and European *B. taurus* dairy breeds combined with *Bos indicus* breeds can significantly increase smallholder incomes (Djoko et al., 2003; Haile et al., 2009; Makoni et al., 2013; SNV, 2013). Documentation of the specific genetic makeup and genetic progress in productivity is, however, limited (Kosgey et al., 2011; Muasya et al., 2013). Smallholder farmers do not keep records on performance, pedigree of animals, or breeding practices (MOLD, 2010; Wambugu et al., 2011). Thus, no specific type of animal has been identified as suitable for smallholders, resulting in farmers rearing a wide range of crossbred animals of varying breed compositions (Muraguri et al., 2004; Muia et al., 2011; Wanjala and Njehia, 2014). This has resulted in a dynamic genetic landscape in which it is not possible to quantify and track the connections between animal and herd performance, between profitability and breeding interventions, and among all 4 factors. An analysis by Rege et al. (2011) concluded that one of the highest priority interventions for smallholder systems is the development of innovative approaches for the strategic use of appropriate genetics from the available range of breed resources, underpinned by a good understanding of existing breed resources that have demonstrated good dairy potential. Lessons learned from nucleus breeding programs in the tropics (Philipsson et al., 2011; Rege et al., 2011; Mueller et al., 2015) indicate the need for a simple approach to data capture from smallholder systems, with tangible incentives for continued recording as a basis for implementation of a sustainable genetic improvement program.

In an attempt to generate baseline information and test the use of genomic information as a tool to guide the breeding of improved dairy cattle for small holder farming systems in East Africa, a project titled “Germplasm for Dairy Development in East Africa” (or Dairy Genetics in East Africa, **DGEA**) was implemented from 2011 to 2013. The project was designed to determine the breed composition and levels of animal performance under different smallholder farm environments to identify the optimum breed combinations for smallholders and then to develop options to sustainably supply the optimal germplasm for improvement of dairy production in the smallholder system (Ojango et al., 2014).

Genetic parameters are fundamental for the development of genetic evaluation systems and therefore the design of any breed improvement program. Currently, in sub-Saharan Africa, estimates of genetic parameters in smallholder systems are lacking primarily because of the lack of suitable data. In this study, using the DGEA data from Kenya, we examined the potential impact of different models handling contemporary groups or herd effects as random or fixed effects on estimates of genetic parameters using a fixed regression model for test-day milk yield. In addition, using a random regression model, we examined the covariance structure for test-day milk yield at various stages of lactation for animals raised on smallholder farms.

## MATERIALS AND METHODS

### Environment and Herd Characteristics

**Figure 1:**
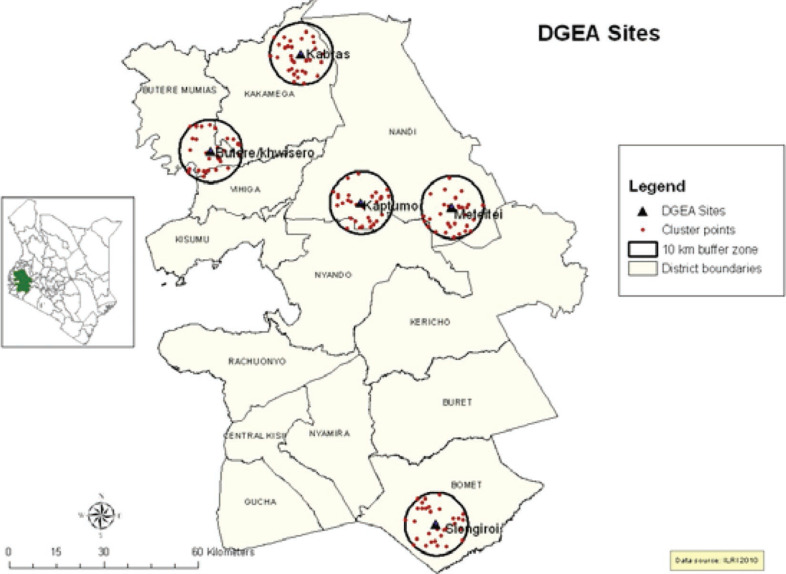
Dairy Genetics East Africa (DGEA) sites in Kenya from which data on smallholder farms were collected.

***Site and Animal Selection Criteria.*** Farm and herd registration, followed by monitoring and cow performance recording over 2 yr, was undertaken by field data collectors with training in animal production who were recruited and trained on dairy cattle data collection by the project. Farms were selected in 5 sites of Kenya that represented a range of climate and farming conditions typical for highland (>1,000 m) subtropical smallholder dairy systems as described in Moll et al. (2007) and Robinson et al. (2011). The majority of livestock were kept under mixed farming systems in small land holdings (<5 ha). The sites comprised districts with a high population of smallholder farmers rearing dairy cattle for milk production and using a variety of genotypes, mostly crossbreds between imported dairy breeds and indigenous types, mainly zebu. Smallholder farmers owned less than 10 acres of land and had fewer than 11 head of cattle. In each site, 31 “clusters” of 5 farmers were randomly identified using global positioning system (GPS) coordinate points (Figure 1). Farms selected for animal recording had 2 or more lactating cows or in-calf heifers characterized either as crossbreds (exotic × indigenous) or pure exotic (based on physical appearance, including coat color and absence of a hump).

***Herd Characteristics.*** Smallholder farm characteristics varied greatly depending on the resource endowment of the farmers. The general practice was to leave the animals to graze in a restricted environment either around the homestead or by the roadside; sometimes the animals were provided with supplementary fodder, typically comprising Napier grass (*Pennisetum purpureum*) and leguminous plants such as calliandra (*Calliandra calothyrsus*) and desmodium (*Desmodium intortum* and *Desmodium uncinatum*), some maize stover, and occasionally some commercial dairy meal. Calves born on the farms were separated from their dams after the first week. The calves were then fed milk using buckets for 4 to 5 mo depending on the farmer’s resource endowment.

### Data

Data were collected on 1,292 cows from 610 farms over 2 yr from January 2011 to December 2012. Data included farm characteristics, herd structure, cattle management practices, access to services, and animal productive and reproductive performance. An animal characterized by the field data collectors as either crossbred or pure exotic had to be lactating or at an advanced stage of gestation to be monitored. Calving dates recorded for lactating animals at the start of the project were based on farmer recall. As monitoring progressed, subsequent dates of calving for different animals were recorded. It was initially not clear whether the parity reported as first parity was actually the first parity of the animal in its lifetime or the first parity of that animal on the specific farm. Each farmer was provided with a jar for measuring milk and a booklet for recording milk produced by individual animals in the morning and in the evening on 1 day within each week of lactation to constitute a test-day (**TD**) yield. The field data collectors visited the farms every 2 wk to measure and verify the quantities of milk produced and recorded by the farmers. To determine the genotypes of the animals on the farms being monitored, hair samples were collected from 1,157 of the crossbred animals and 212 reference animals that were believed to be pure indigenous.

Data from animals sampled for genotyping with information on calving dates and a minimum of 4 TD records over at least 2 mo within a lactation were used in this study. In the course of monitoring, some farmers withdrew from the project and some animals exited farms, leading to incomplete data. Information on the age of animals was not comprehensive. The parity reported for an animal was used as a proxy for age because estimating age based on the number of incisor teeth and their level of wear was not implemented consistently across sites. The DIM was calculated as the number of days the animal had been milking from the day it calved to the day the milk was recorded by the field data collector.

The TD records were subjected to various tests for accuracy and consistency before analyses. Using dates of calving based on farmer recall for animals that were already in milk, the DIM for some animals were very long (up to 550 d). The TD records were thus grouped into 100-d stages and evaluated using linear regression (Ojango et al., 2014). Through these analyses, it was evident that more than 80% of the TD records were within 310 DIM (lactation lengths ranged from 250 to 550 d). Out of 31,657 TD records, a final data set of 21,877 TD records from 1,038 animals in 566 herds were used to evaluate the 305-d milk production of animals of different breed-types on smallholder farms. Lactation length was set at 305 d in line with the worldwide practice of evaluating dairy cattle on a 305-d lactation period.

### Classification of Animals into Breed Groups Using Genotypic Information

Animals were genotyped using the 777k Illumina Bovine BeadChip (Illumina Inc., San Diego, CA), as outlined in Ojango et al. (2014). The resultant SNP data were subjected to principal components analyses and analyses to obtain estimates of breed composition using the Admixture software (Alexander et al., 2009). Five exotic “dairy” breed types, Ayrshire, Guernsey, Holstein, Friesian, and Jersey, and 2 indigenous breed types, Nelore and N’Dama, were used as ancestral breeds in the analyses. Nelore and N’dama were used as proxies for pure *B. indicus* and African *B. taurus* because prior analyses have shown that East African indigenous breeds are ancient hybrids of *B. indicus* and African *B. taurus* cattle (Weerasinghe et al., 2013; Strucken et al., 2017). The combined proportions of each animal’s exotic dairy breed alleles obtained from the Admixture analyses were used to group animals into 5 breed classes (percent dairy group) where the exotic dairy contribution ranged from 0 to 20% (group 1), 21 to 35% (2), 36 to 60% (3), 61 to 87.5% (4), and >87.5% (5). Estimates of individual dairy breed contributions to each animal’s genotype (e.g., proportion of Friesian vs. Holstein vs. Ayrshire) were more dependent on the analyses performed and are not reported here.

### Defining Contemporary Groups

Following the conventional practice for the analysis of dairy cattle data, contemporary herd groups that consisted of herds (**H**) or herd-year-season (**HYS**) were fitted as either random or fixed. Four seasons were defined within each year as green or dry based on the rainfall pattern of the sites (Stotz, 1983) as December to March (dry season 1), April to June (green season 1), July to August (dry season 2), September to November (green season 2).

Given the small herd sizes, 2 other definitions of contemporary herd groups were examined. First, using principal components analysis, we estimated the proportion of variance contributed by different variables (land size, region, feeding, and marketing system). This was followed by optimal extraction of number of principal components using K-means selection. Extracted factors were then used for discriminant analysis of the principal components (DAPC), leading to posterior predictions for group membership for each household using the R statistical package (Jombart and Ahmed, 2011). The herd groups (**HG**) obtained are presented in Table 1. Second, we grouped herds based on solutions for random herd effects from a mixed-effects regression model, as follows:

**Table 1:** Definition of herd management groups (HG) and the number of herds in each group

Herd management group	Characteristics	Total households (no.)
1. High-input intensive production system	Smallholder intensive, supplement heavy, high feed mix, commercially oriented dairy production system	341
2. Medium-input improvised dairy production system	Mixed improvised dairy production system, moderate labor input, commercially oriented	54
3. Extensive production system	Small stock extensive dairy, low labor input, low supplementation, large land area, low feed mix, production system	323

y = Xb + Qu + Wsf + e, [1]

where **y** = individual test-day yields, and **b** = solutions for the fixed effects in the model, with the following fixed effects: year-season of TD *j* (*j* = 1–10), parity *k* (*k* = 1, 2, ≥3), percent dairy group *l* (*l* = 1 to 5); lactation stage in 100-d intervals *m* (*m* = 1 to 4), **u** = random cow effects, **sf** = random herd effects, **e** = random residual effects, **X** is the incidence matrix relating records to fixed effects, and **Q** and **W** are incidence matrices that relate records to cows and farms. The variance of random cow effects, $\mathrm{var}\left(u\right)\;=\;\sigma_{u}^{2}$, variance of random herd effects, $\mathrm{var}\left(sf\right)\;=\;\sigma_{sf}^{2}$, and the residual variance equal $\mathrm{var}\left(e\right)\;=\;I\sigma_{e}^{2}$, where **I** is the identity matrix. Solutions for random herd effects from this model were ranked into bottom, middle, and top to create 3 herdlevel (**HL**) classes. This approach was taken so that the data defined the farm production environment experienced by the cow; in essence, the association of animals in herd groups depended on their level of milk production and was not random. Initial results from this analysis were presented in Ojango et al. (2014).

### Estimation of Genetic Parameters with Different Models

Genetic parameters were estimated using a genomic relationship and a fixed regression model (**FRM**) fitting several models. Various analytical models were considered to explain the shape of the lactation curves iteratively. Preliminary data analysis using various lactation functions gave the Legendre polynomial as the best fitting curve. The order of polynomials used in this study was the best possible in terms of being able to achieve convergence and ensure that a curve with at least 3 parameters was fitted for the random animal component. A higher order for the animal component did not converge because of inadequate information, and a lower order was inferior.

A quadratic regression on Legendre orthogonal polynomials of DIM was used to model the mean trend. Analyses were implemented using ASREML (Gilmour et al., 2009).

The general FRM was

$$y_{tij\;}=Fixed_{i}+\sum_{k=0}^{4}\varphi_{tjkm}\beta_{km}+u_{j}\;+\;pe_{j}\;+\;e_{tij},$$ [2]

where *y_tij_* is the TD record of cow *j* on day *t* (DIM 1–305); *Fixed_i_* are the *i*th fixed effects consisting of contemporary herd group (defined as H, HG, or HL), yearseason of test-day, parity group, percent dairy (Table 2); *β_km_* are *k*th fixed regression coefficients for percent dairy nested within a contemporary herd group (when HG or HL was included); **φ***_tjkm_* is the vector of the *k*th Legendre polynomials (*k* = 1–4), for the TD record of cow *j* on day *t*; **u***_j_* and **pe***_j_* are vectors of animal additive genetic and permanent environmental effects, respectively, for animal *j*; and *e_tij_* is the random residual. It was assumed that $\mathrm{var}\left(u\right)\;=\;G\sigma_{a}^{2},\;\mathrm{var}\left(pe\right)\;=\;I\sigma_{p}^{2},$ and $\mathrm{var}\left(e\right)\;=\;I\sigma_{e}^{2}\;=\;R$, where **I** is the identity matrix and **R** is the matrix of residual variances. Matrix **G**, the genomic relationship matrix, was constructed from the genotype file consisting of 745,059 SNP as in Brown et al. (2016). The SNP used in the construction of **G** were those remaining after applying edits to the original 777k SNP obtained using the Illumina high-density BeadChip for genotyping the cows.

**Table 2:** Factors influencing test-day (TD) milk production, number of TD records, and TD milk yield (LSM, SE in parentheses) at each factor level for smallholder farms

Variable, significance, and level	No. of TD records	TD Milk yield
Herd environment level (HL)		
1	8,361	3.50 (0.07)
2	9,157	4.67 (0.07)
3	7,601	6.83 (0.07)
Herd management group (HG)^***^		
1. High input	17,153	5.18 (0.07)
2. Medium input	1,628	4.69 (0.12)
3. Extensive	6,338	4.74 (0.08)
Year-season of test-day^***^		
10	25,119	5.20 (0.25)
Parity group^***^		
1	15,464	4.79 (0.06)
2	9,655	5.35 (0.06)
Percent dairy^***^		
0–20%	246	4.80 (0.23)
21–35%	669	4.61 (0.14)
36–60%	4,543	4.44 (0.07)
61–87.5%	11,106	4.98 (0.06)
>87.5%	8,555	5.52 (0.06)

*** *P* < 0.001.

The matrix **G** was constructed using VanRaden’s first method (VanRaden, 2008) as

$$G\;=\;\frac{ZZ^{\prime }}{2\sum p_{i}\left(1-p_{i}\right)}$$ [3]

where **Z** is a design matrix of centered genotypes, and *p_i_* is the allele frequency estimated across breeds for the major allele at SNP *i*.

Using the basic model presented in equation [2], different models were examined:

- model 1: excluding the effect of herds, assuming the animals were random in a smallholder system;
- model group 2: including a random effect of herd (H), either with or without groups for percent dairy;
- model group 3: including a random herd-yearseason (HYS), with or without groups for percent dairy;
- model group 4: including herds as a contemporary group defined using HG, with or without the percent dairy;
- model group 5: including herds as a contemporary group defined using HL, with or without the percent dairy.

The different FRM models were compared using the Akaike (**AIC**) and Schwarz’s Bayesian (**BIC**) information criteria (Wolfinger, 1993).

The heritability estimate for TD from each FRM was obtained as

$$\sigma_{a}^{2}/\left(\sigma_{a}^{2}\;+\;\sigma_{p}^{2}\;+\;\sigma_{e}^{2}\right).$$ [4]

The best FRM identified using AIC and BIC was then used to compute lactation curves for animals in the contemporary groups defined by the model, and to model the covariance structures among the test-day yields using random regression analyses (**RRM**). The RRM fitted was

$$y_{tij}\;=\;Fixed_{i}\;+\;\sum_{k=0}^{4}\varphi_{tij}\beta_{km}\;+\;\sum_{k=0}^{2}\varphi_{tjk}u_{kj}\;+\;pe_{j}\;+\;e_{tij},$$ [5]

where terms were as defined in equation [2] but **u***_kj_* here is a vector of *k*th random regression coefficient for cow *j*. The same structure was fitted for permanent environment (**pe**) effects because there was a convergence problem with fitting a higher order of equations for this effect due to the limited data size. Different residual variances associated with different stages of lactation (stage 1 = 1–60; 2 = 61–120; 3 = 121–200, and 4 = 200–305 DIM) were fitted. The var(*pe*) was defined as in equation [2] and var(*u*)=**GM**, where **M** is the genetic covariance of order 3 between the random regression coefficients.

The genetic variances (var*_gii_*) of a given DIM (**t***_i_*) and the genetic covariances (cov*_gij_*) between different DIM (**t***_i_* and **t**′*_i_*) were obtained from the RRM analysis as ${\mathrm{var}}_{gii}\;=\;t_{i}M{t^{\prime }}_{i}$ and ${\mathrm{cov}}_{gij}\;=\;t_{i}M{t^{\prime }}_{j}$, respectively, where **M** is the co-variance matrix obtained from the ASREML analysis. The phenotypic (co)variance was calculated as the sum of the genetic, permanent environmental, and residual (co)variances. The estimate of heritability for day *i* was obtained as ${\mathrm{var}}_{gii}/\left({\mathrm{var}}_{gij}+\sigma_{p}^{2}+\sigma_{e}^{2}\right),$ and the additive genetic correlation (*Cor_a_*) between days *i* and *j* was obtained as

$$Cor_{a_{ij}}\;=\;\frac{\sigma_{a_{ij}}}{\sqrt{\sigma_{a_{i}}^{2}\;+\;\sigma_{a_{j}}^{2}}}.$$ [6]

## RESULTS

### Summary of TD Milk Production Performance

Summary statistics from the least squares analyses of variance for the TD data with the environmental factors fitted in the model are presented in Table 2. About 95% of the herds in the analyses had 1 to 3 animals in milk. Overall, 46.6% (484) of the animals had milk recorded in 1 lactation, whereas 53.4% (554) had milk records from 2 lactations. However, only 8.6% of the animals with milk records in a second lactation had records covering more than 5 mo in the second lactation. The average TD milk production for all the animals raised on smallholder farms was 5.38 ± 3.23 kg. The coefficient of variation was large (>60%), indicating a wide range in TD milk values.

Consistent with the results of Ojango et al. (2014), animals raised in high-input systems (HG-1) had a higher overall TD milk yield than those raised under lower-input systems. The TD milk production levels for animals in the 3 herd groups obtained from assigning the animals to different productivity levels (HL) were also significantly different (Table 2). Additionally, animals whose genetic make-up comprised >87.5% exotic breed-type (i.e., percent dairy) had a higher mean TD milk yield than those with a lower proportion of exotic breed-type. We also detected significant differences in TD milk production over different seasons within years when milk was recorded. The TD milk production was highest in the first green season of each year, in which there is usually more rainfall, ranging from (LSM with SE in parentheses) 5.37 (0.07) to 6.09 (0.24) kg compared with the other seasons of the year, which ranged from 4.21 (0.12) to 5.98 (0.07) kg.

### Parameter Estimates for Models with Different Contemporary Herd Groups

Genetic parameter estimates obtained from the different FRM are presented in Table 3. The highest estimate for heritability was obtained when the effect of the herd was not included in the analytical model (0.27 ± 0.06, model 1). However, this model yielded the worst fit (AIC and BIC), indicating that even with very small herds, variances differ across herds and need to be accounted for in the analyses. In contrast, including herd as a random effect resulted in a large proportion of the variation being attributed to the herd and very little to the individual animals (model group 2); residual variance, however, remained the same as for model 1.

**Table 3:** Parameter estimates from fixed regression analytical models and the criteria of information of Akaike (AIC) and Schwarz’s Bayesian information criteria (BIC) for fixed and random regression models

				Variance ± SE		
Analytical models^1^: Contemporary herd grouping	AIC^2^	BIC^2^	Heritability	Permanent environment	Random herd	Residual
Fixed regression models						
Model 1: None, without percent dairy	73,002	73,026	0.27 ± 0.06	0.17 ± 0.05		0.56 ± 0.013
Model group 2: Random herds (H)						
Without percent dairy	72,852	72,884	0.07 ± 0.03	0.13 ± 0.03	0.24 ± 0.02	0.57 ± 0.014
With percent dairy	72,783	72,816	0.05 ± 0.03	0.14 ± 0.03	0.24 ± 0.02	0.57 ± 0.014
Model group 4: Herd management (HG)						
Without percent dairy	73,001	73,026	0.26 ± 0.06	0.18 ± 0.05		0.56 ± 0.013
With percent dairy	72,913	72,938	0.27 ± 0.05	0.17 ± 0.05		0.56 ± 0.013
Model group 5: Herd environment (HL)						
Without percent dairy	72,482	72,506	0.10 ± 0.04	0.20 ± 0.04		0.70 ± 0.011
With percent dairy	72,278^3^	72,303^3^	0.094 ± 0.04	0.21 ± 0.04		10.70 ± 0.011
Random regression model: HL, with percent dairy	58,787	58,883				

1 See text for description of models.

2 AIC = −2log likelihood + 2*p*; BIC = −2log likelihood + *p*log[N − r(x)], where *p* is the number of estimated parameters, N is the sample size, and r(x) is the matrix rank of the fixed effect coefficients in the analysis model.

3 Indicates the best fixed regression model based on AIC and BIC.

Analytical models where the herd groups were defined using production levels derived from the data (HL) yielded a better fit than when herds were grouped depending on management practices (HG) or were random. Residual variance was also higher when herd groups were defined using HL. Inclusion of herd grouping based on the adoption of different management practices (HG) resulted in a substantially higher heritability estimate and slightly lower permanent environmental variance than when herds were grouped by HL (Table 3). Inclusion of percent dairy in the models resulted in a better fit based on the AIC and BIC; however, it did not significantly affect the heritability estimates in any of the models. Models including a random HYS effect (model group 3) failed to converge; hence, no results are presented.

**Figure 2:**
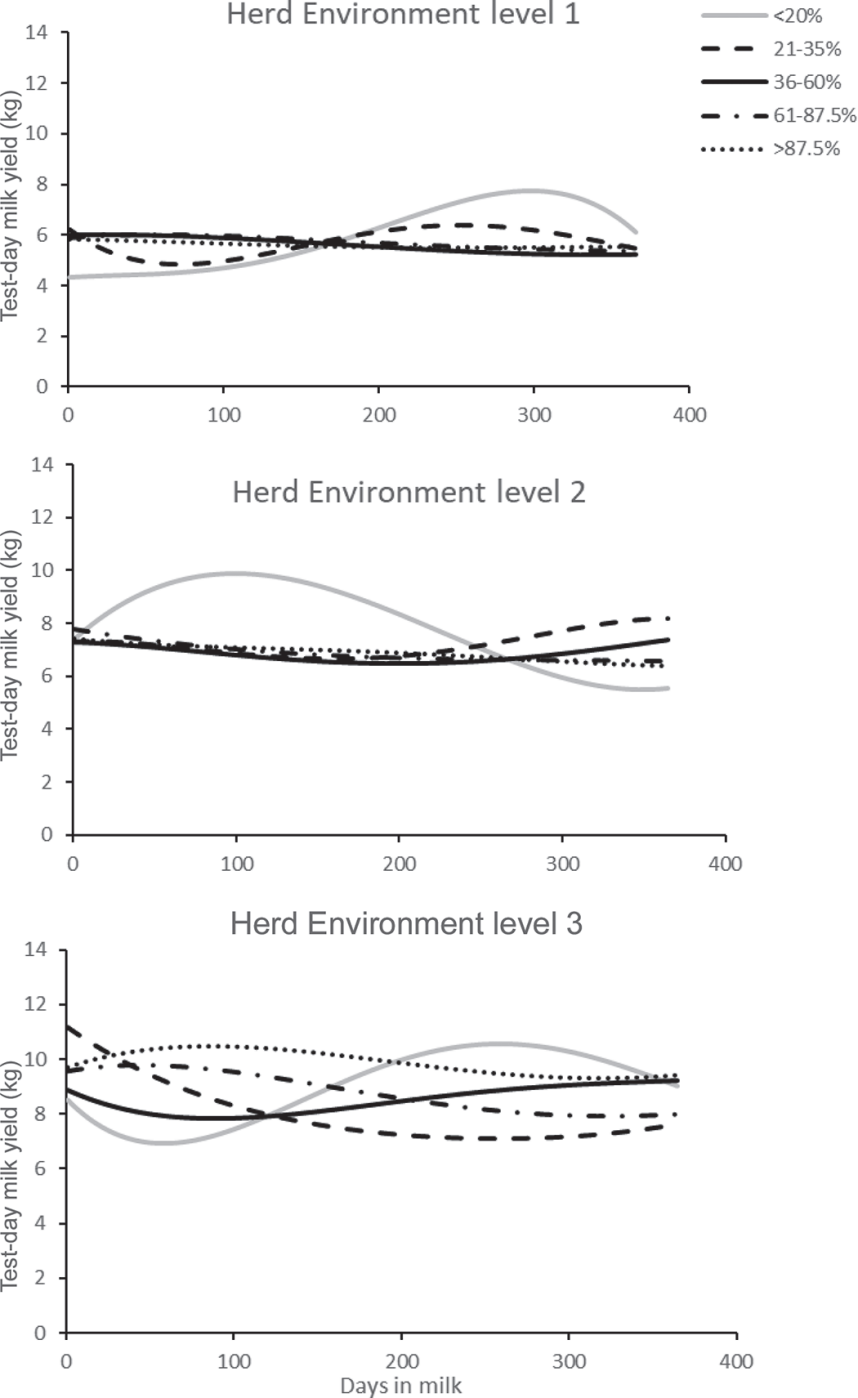
Lactation curves for different breed-groups of animals with different percentages (<20 to >87.5%) of exotic breed-types (i.e., percent dairy) under the different herd environment groups (levels 1–3) on smallholder farms in Kenya.

The lactation performance of animals with different percent dairy derived from the best FRM is presented in Figure 2. As anticipated from the grouping by HL, the average productivity of animals with different proportions of exotic genotypes was highest in the better herd environment level (HL-3). Within HL-3, animals with a high proportion of exotic genes (>87.5% dairy) were most productive. In the moderate herd environments, HL-2 (animals with a lower proportion of exotic genes) performed better than all the other breed types.

### Genomic EBV for Animals with Different Proportions of Exotic Breed-Types

**Figure 3:**
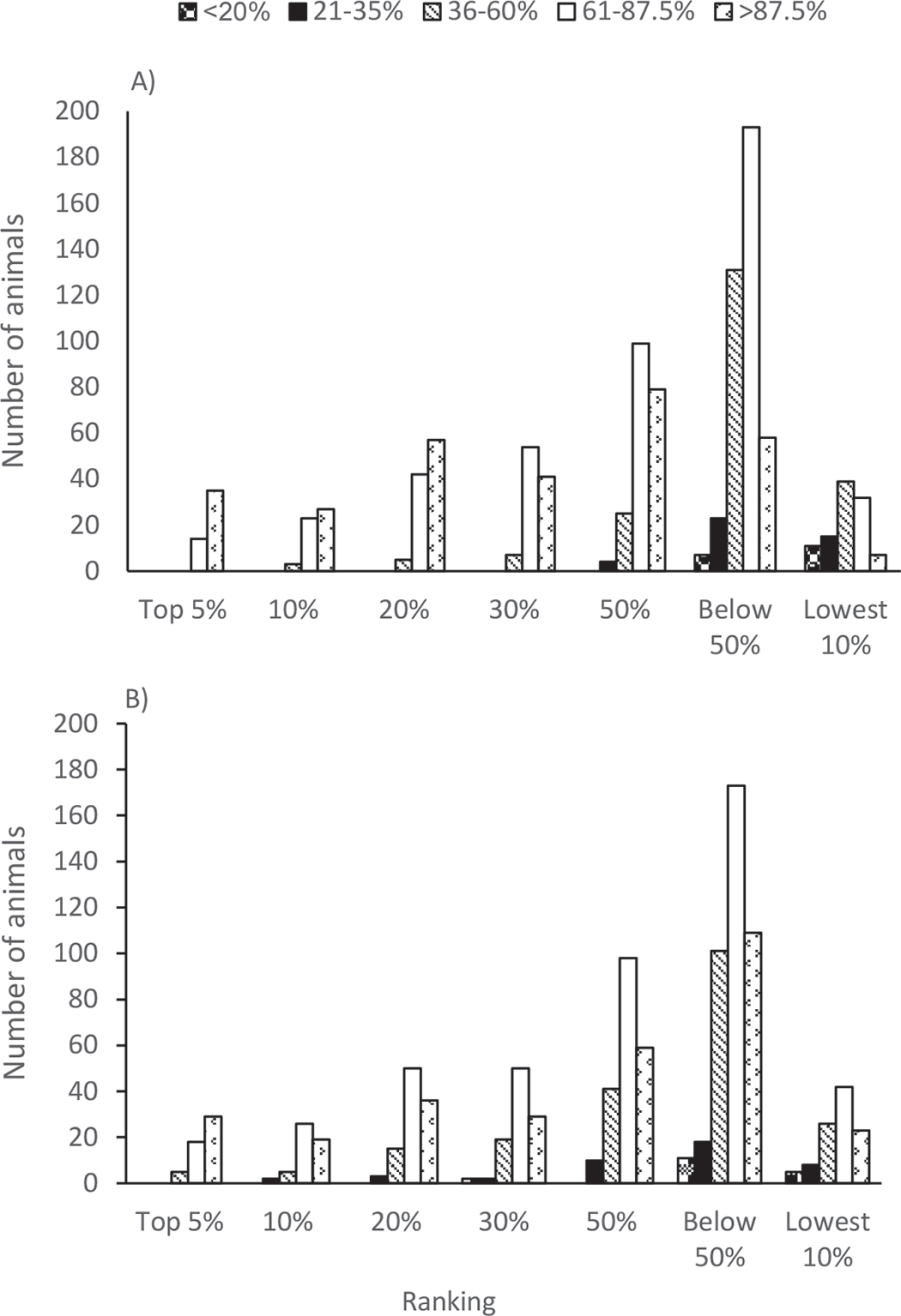
Genomic EBV rank categories for animals with different proportions of exotic breed types (i.e., percent dairy). (A) No % dairy included in the model, and (B) % dairy included in the model.

The genomic (g)EBV ranks for animals with different percent dairy from the 2 best FRM models are presented in Figure 3. The gEBV from the model that included percent dairy resulted in cows ranked in the top 5 and 10% consisting of cows with exotic genes varying from 36 to 87.5%, and with the top 30% including cows with 21 to 35% exotic genes. Cows with 87.5% exotic genes were highest in the top 5 to 30%. When percent dairy was not included in the analytical model, the animals ranked among the top 5% included only cows with exotic genes >61%, and the top 30% never included cows with <36% exotic genes. This result demonstrates the need to include percent dairy in the model.

### Variance Component Estimates from Random Regression Analysis

**Figure 4:**
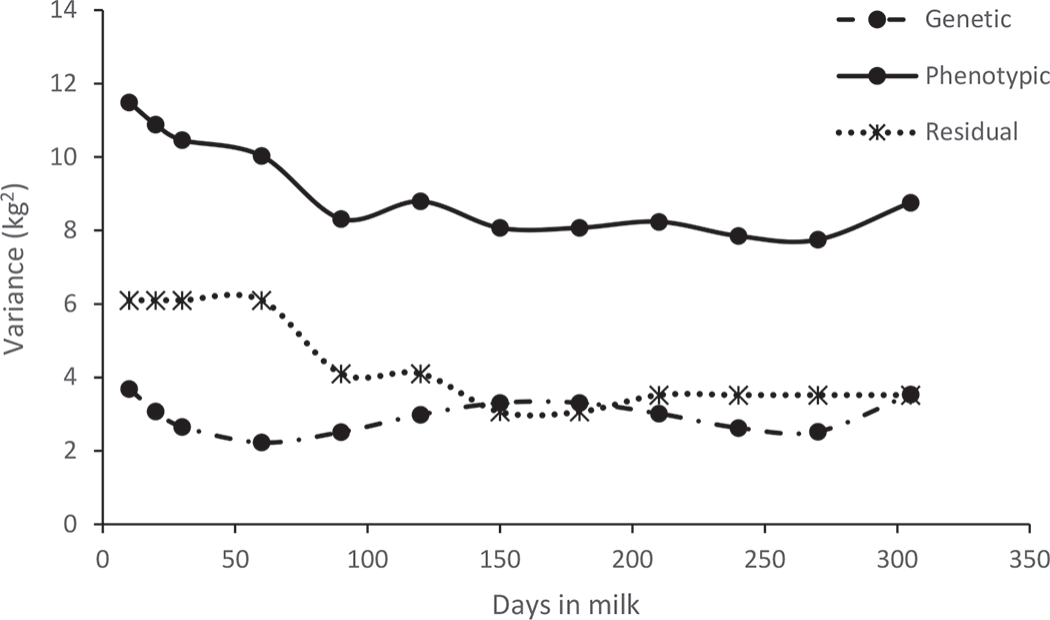
Estimates of genetic, phenotypic, and residual variances (kg^2^) for test-day milk yield over 305 DIM.

The analysis using the RRM fit the data better than the FRM models based on the AIC and BIC (Table 3). Estimates of genetic and phenotypic variance for TD milk in the smallholder herds using the RRM are presented in Figure 4. Phenotypic variance was high during the first month of lactation and then declined in the second month. Genetic variance also declined from the first to second month but increased slightly in mid lactation. When the approximate heritability for the whole lactation (305 d) was obtained as the heritability of the average 305-d TD yield for the days in Table 4, the heritability was 0.19.

**Table 4:** Heritability estimates (diagonal), genetic correlations (below diagonal), and phenotypic correlations (above diagonal) between test-day milk records from smallholder farms in Kenya

DIM	10	20	30	60	90	120	150	180	210	240	270	305
10	**0.32**	1.00	0.99	0.92	0.72	0.62	0.48	0.42	0.42	0.39	0.37	0.33
20	0.99	**0.28**	1.00	0.95	0.76	0.68	0.54	0.48	0.48	0.45	0.41	0.35
30	0.96	0.99	**0.25**	0.97	0.80	0.73	0.59	0.54	0.54	0.50	0.45	0.37
60	0.71	0.81	0.89	**0.22**	0.87	0.83	0.73	0.68	0.67	0.62	0.55	0.41
90	0.39	0.52	0.64	0.92	**0.30**	0.99	0.89	0.86	0.85	0.79	0.69	0.50
120	0.14	0.27	0.42	0.79	0.96	**0.34**	0.93	0.91	0.91	0.85	0.74	0.53
150	−0.05	0.08	0.23	0.65	0.89	0.98	**0.41**	0.99	1.00	0.94	0.84	0.62
180	−0.21	−0.07	0.07	0.51	0.80	0.93	0.98	**0.41**	1.02	0.98	0.89	0.69
210	−0.35	−0.23	−0.09	0.35	0.66	0.83	0.92	0.98	**0.37**	0.98	0.92	0.75
240	−0.48	−0.38	−0.27	0.13	0.45	0.65	0.78	0.88	0.96	**0.33**	0.97	0.85
270	−0.57	−0.52	−0.45	−0.17	0.11	0.32	0.48	0.62	0.77	0.92	**0.33**	0.95
305	−0.52	−0.54	−0.55	−0.49	−0.33	−0.16	−0.01	0.16	0.36	0.61	0.87	**0.40**

The heritability estimates (h^2^) for specific TD yields obtained by the RRM were higher than those obtained by the FRM (Table 4). The estimates obtained were lower in the first 3 mo of the lactation (DIM 20–90). The phenotypic correlation estimates were all positive, ranging from 0.33 to 1.00. Higher values were observed between adjacent TD and lower values were observed between TD at the beginning and at the end of the lactation. The phenotypic correlations were generally larger than the genetic correlations.

Genetic correlation estimates ranged from −0.01 to 0.99. The correlations were high and positive for measures occurring within short consecutive intervals but decreased as the intervals between the TD increased beyond 60 d. Negative genetic correlations were obtained between the first TD and TD after the mo 5 of lactation, between the second TD and TD after mo 6 of lactation, between the third TD and TD after mo 7 of lactation and continued in a similar pattern over the lactation. Estimates of the variance and covariance components between random regression coefficients are presented in Table 5. The correlation between the lactation average (related to milk yield) and slope (related to the rate of decrease in milk yield) was negative. The change in sign suggests contrasting effects on milk yield at the beginning and later in lactation. Cows that start their lactation at a lower TD yield have a slower rate of reduction in milk yield and thus higher persistency than cows that start their lactation at a higher TD yield. Partitioning the residual variance for different stages of lactation in the analyses improved the model but did not change the negative genetic correlations.

**Table 5:** Estimates of variance (diagonal) and covariance (below diagonal) between random Legendre polynomials

Polynomial	1	2	3
1	**2.041**		
2	0.243	**3.14**	
3	−1.01	0.83	**1.83**

## DISCUSSION

### TD Milk Production Levels Under Smallholder Farming Systems

Results from our study show a low level of milk production for dairy cattle with various proportions of exotic genotypes raised under smallholder farming conditions. The TD milk production per cow in this study, although similar to that reported for smallholder systems in Kenya (Muraguri et al., 2004; Wanjala and Njehia, 2014), was lower than yields reported for *B. indicus* and *B. taurus* crosses in Brazil (Pereira et al., 2013) but higher than the yields reported for similar crosses in Eastern Africa (Galukande et al., 2013). The productivity exhibited by the more exotic genotypes was well below the potential productivity of these animals when raised under better production conditions, even within East Africa (Ojango and Pollott, 2001; Wasike et al., 2011). A meta-analysis of published information on dairy production in Africa by Mwacharo et al. (2009) found a difference in milk production levels attainable under smallholder farming systems relative to largerscale farming systems of up to 75% that was attributed to differences in production and management practices adopted by livestock keepers in low-income countries.

The lactation curves obtained for animals raised under the different production categories (HL) for the smallholder farmers did not display the typical peak yield in the first months of the lactation followed by a gradual decline (Figure 2). Only in improved smallholder herd environments (HL-3) were animals with a higher proportion of exotic genotypes able to exhibit their higher milk production potential relative to animals with a higher proportion of indigenous genes. Under medium- and large-scale farming conditions in Kenya, the lactation curves for dairy cattle have displayed a peak yield within the first 2 mo of lactation (Ojango and Pollott, 2001; Wasike et al., 2011). The large variation in milk production exhibited by animals within the smallholder systems in this study presents an opportunity for a targeted selection program to improve milk production using the existing breed types.

### Effects of Different Contemporary Herd Groups

Results from the FRM analyses illustrate how the magnitude of the parameter estimates is highly dependent on the model used. Theoretically, inclusion of the herd as a random effect for multiple smallholder farm environments should maximize the effective number of records for evaluation and increase the accuracy of breeding values. However, with the small herd sizes in this data set, it was likely difficult to separate the random herd effect from the random animal genetic and permanent effects, and the errors attached to parameter estimates may be substantially underestimated. Nevertheless, it is intriguing that the estimate of permanent environment variance was little affected by fitting a random herd variance, whereas the estimate of additive genetic variance was substantially reduced.

Fitting HG as the contemporary group, although statistically significant as a fitted effect, had almost no effect on AIC and BIC and had a trivial effect on estimates of variance (model group 4 vs. model 1 in Table 4).

When percent dairy was added to the models, the statistical fit was improved substantially but the estimates of variances were hardly affected. Genetic parameter estimated obtained from model groups 1 and 4 were within the range of estimates obtained for milk production from studies on *B. indicus* and *B. taurus* cattle in Ethiopia (Demeke et al., 2004; Haile et al., 2009). However, when herds were grouped depending on their level of production (HL), which followed a 2-step analyses to define contemporary herd groups, model group 5 yielded a substantially better fit based on the AIC and BIC (Table 4). The odd feature of these models, however, is that the estimate of residual variance was about 25% higher than for all previous models. The increase in residual variance was accompanied by a large decrease in genetic variance, with the estimates of heritability being much closer to those with herd fitted as a random effect than to other models. We note that whether or not random herd effects are absorbing some of the true genetic variance between animals, HL is expected to have a similar, albeit reduced, effect on estimated genetic variance because it is derived from random herd effects in a 2-step process. It is unclear, however, why the variance between herds that is not explained by HL flows into the estimate of residual variance rather than the genetic variance, as is the case for models 1 and 4.

Studies that quantify the impact of herds in genetic analyses of smallholder farms are limited. In a simulation study, Powell et al. (2018) reported higher accuracies in EBV when herd was fitted as a random effect for herds with more than 4 animals. However, when herds had 1 or 2 animals, they reported that accuracies of models including the herd as a random effect were comparable to those that excluded the herd effect. However, in that study, it was assumed that animals were randomly assigned to herds and hence there was no confounding between herds and animal effects, whereas in our data, there was confounding of herd and animal effects because genetic relationships within herds were higher than those between herds (results not shown). Given the limited data size, it remains unclear whether the realized heritability in these smallholder systems is actually quite low, as inferred when fitting random herd effects, or very substantial, as when fitting models not including or based on random herd effects. The ongoing “Platform for African Dairy Genetic Gains” (ADGG; ILRI, 2018) project, with data being collected on several thousand cows in smallholder farming systems, might help in better understanding estimates of genetic variation in small holder systems.

### Parameter Estimates from RRM

The heritability estimates in this study were higher from the RRM than from the FRM (Table 4). The heritability estimates tended to be lower in the first 60 d of the lactation and were highest in mid lactation (150–210 DIM), mainly because of the lower residual variances (Figure 4). The heritability estimates obtained were higher than those reported in crossbred *B. taurus* × *B. indicus* cattle in Brazil (Bignardi et al., 2009; Pereira et al., 2013), for *B. indicus* cattle in Kenya (Ilatsia et al., 2007), and from other studies for *B. taurus* animals raised under tropical environments (Rekaya et al., 1999; Druet et al., 2003; Muasya et al., 2014). The magnitude of the heritability estimate implies an opportunity for a reasonable response to selection for milk production within the dairy cattle population currently on smallholder farms.

Evaluating parameters over the course of lactation using an RRM enabled a better understanding of the changing influence of the environmental factors over the lactation period. For example, soon after calving, nongenetic factors associated with management practices (including farmer decisions on drying off animals in the previous lactation as well as the absence or presence of “steaming up” before calving) strongly influence milk production, potentially explaining the higher residual variances in early lactation. In the smallholder farming systems of this study, many farmers continued to milk their animals until the last month before calving, even though production levels were low (0.5 kg). This practice negatively affects milk production in the subsequent lactation.

Genetic correlations between TD yields decreased as the time between the TD measures increased. Negative genetic correlation estimates between early and later TD records, as obtained in this study, have been reported in studies on dairy animals raised in tropical environments (Rekaya et al., 1999; de Melo et al., 2007; Bignardi et al., 2009). Correlations among TD yields have also been shown to decrease with distance between TD measures in studies on animals raised under better production environments (Rekaya et al., 1999; Druet et al., 2003). The negative genetic correlation could also be influenced by fewer animals (474) with TD milk yields toward the end of the lactation (250–305 DIM). The magnitude of the genetic correlation estimates among TD records obtained in this study indicates that TD milk records beyond a 60-d interval should not be used as repeated measures of the same trait for genetic evaluation of animals on smallholder farms. Use of a few widely spaced TD records in smallholder systems is also not optimal. However, with the high genetic and residual correlations between TD of adjacent months, recording of milk production for animals raised on smallholder farms could be undertaken once every 1 to 2 mo as proposed for India (Duclos et al., 2008), where herd sizes are small and information is collected through technicians.

Patterns of phenotypic variances across lactation stages similar to those in this study have been reported for both crossbred *B. taurus* × *B. indicus* cattle of Brazil (Pereira et al., 2013) and for purebred *B. taurus* animals raised under tropical production systems (Bignardi et al., 2009).

The results from this study show that in Kenya, smallholder farmers—through a combination of natural and artificial selection pressures—have retained dairy cows of mixed breeds with different levels of various exotic breed-types. The very small herd sizes for milking animals (1–3 animals) and the lack of pedigree and performance data on animals in smallholder farming systems have restricted their genetic evaluation in the past. Selection and breeding for improved milk production in the herds is not practiced. The lack of an organized selection program for dairy production under the smallholder farming system leads to random fluctuations in the genetic level of the population and a general reduction in productivity of the dairy sector. With the ancestral breed composition of each animal determined through Admixture analysis combined with the use of the genomic relationship matrix, it was possible, for the first time, to implement a genetic evaluation of the dairy population from different smallholder farm environments using accurate information on their breed composition. Our results are supported by a simulation study on the use of genomic BLUP for genetic evaluation of phenotypic data from smallholder farming systems (Powell et al., 2018). That simulation study reported EBV accuracies for animals with records of >50% at low levels of genetic connectedness with 4 offspring per sire in 2,000 and 4,000 herds.

Although individual smallholder farmers keep only a few animals, the large number of farmers operating under specified environmental parameters provide a sufficiently large herd-group for a breeding program. Advances in mobile phone technologies can facilitate farm data collection. In addition to milk production, information is required on other adaptive traits and non-income functions of the cattle reared. Different “dairy” lines could be developed for different smallholder environments. To obtain accurate information on the dairy cattle within smallholder production systems, longer-term monitoring of the livestock population is required.

## CONCLUSIONS

Results from this study highlight the impact of alternative definitions of contemporary herd groups on genetic parameter estimates. The optimal contemporary herd group definition depends greatly on individual smallholder herd size and the overall sample size of the data used for analyses. Although each individual smallholder farmer retains only a few animals, the large number of farmers operating under specified and often similar environments provides a sufficiently large herd-group for which a breeding program could be developed. Genetic correlations between consecutive TD measures with intervals <60 d were high and positive and then decreased as the TD interval increased, implying that in the smallholder systems studied, TD milk data for genetic evaluation of animals should not be recorded at intervals >60 d for adequate modeling of lactations. The study demonstrated that estimation of genetic parameters, and therefore genetic predictions, are feasible in smallholder systems using the **G** matrix in the absence of pedigree information.
